# Supplementary material for: “It’s a habit. They’ve been doing it for decades and they feel good and safe.”: A qualitative study of barriers and opportunities to changing antimicrobial use in the Indonesian poultry sector
Source: PLoS One. 2023 Sep 25;18(9):e0291556. doi: 10.1371/journal.pone.0291556 (PMC10519599; doi:10.1371/journal.pone.0291556)
Supplement: S1 Table — (PDF) [file pone.0291556.s003.pdf]

Supplementary Table S1: Interviewees' opinions on the impact on production and profitability of the 2018 ban on antibiotic growth promoters in livestock*

Type of impact	Excerpt	Interviewee
Reduced production and/or increased costs	“The most severe outbreak was around 3 years ago, when we are no longer allowed to use antibiotic growth promoters that actually really influenced performance in our, in industry.”	ASSOCIATION_02
	“That [impact of the ban] can be up to 20% -30% down in production, very drastic.”<br>“Ok, yes, that's the problem. If you don't use AGP, production will drop.”	OTHER_02
	“So the cost of production, then the results of production, that's what burdens the breeders when the majority are asked about the AGP ban.”	OTHER_06
	“Yes, performance has decreased, mainly since the implementation of [the ban in] broiler farms.”	OTHER_10
	“The mortality when we stop using the AGP, the mortality was quite increase at that time, I think the average about from, average four percent up to six or seven percent at the time […] And also the effect of banning the AGP, the length of production time is more time.”	OTHER_17
Increased disease	“I think the challenge is higher. After not using it [antibiotics], the disease challenge is higher. Yes, even though the aim is good when it comes to eliminating antibiotics, but the maintenance is difficult.”	OTHER_07
No impact overall	“In 2018, actually, we didn’t use that [AGP] because it was government policy […] so the cost for production would actually increase, and with this increasing cost, it would reduce profitability. However, on the other hand with this, well actually [the chickens are] growing slower, so the meat producing would be also smaller, so we have two things in 2018. So because the supply becomes lower, then the price actually increases, the price skyrocketed, and at that time in terms of profit, the performance was bad, but profit is actually relatively unaffected.”	ASSOCIATION_02
	“On the production impact, it [impact of the AGP ban] is not visible.”	FARMER_07
	“It [impact of the AGP ban] doesn't look too different I feel. It looks like there's no problem.”	FARMER_12
Improved production and/or reduced costs	“We didn’t have problem since the change of year in 2018. I saw that we have best performance, in my opinion.”	MANAGER_01
	“Only the health of the chicken is better. Secondly, it [impact of AGP ban] saves money. The cost savings are extraordinary.””	FARMER_07

* AGP = antibiotic growth promoters, AMU = antimicrobial use.
