# Supplementary material for: “It’s a habit. They’ve been doing it for decades and they feel good and safe.”: A qualitative study of barriers and opportunities to changing antimicrobial use in the Indonesian poultry sector
Source: PLoS One. 2023 Sep 25;18(9):e0291556. doi: 10.1371/journal.pone.0291556 (PMC10519599; doi:10.1371/journal.pone.0291556)
Supplement: S2 Table — (PDF) [file pone.0291556.s004.pdf]

Supplementary Table S2: Interviewees' opinions on the impact on AMU and AMR of the 2018 ban on antibiotic growth promoters in livestock*

**Type of impact**	**Excerpt**	**Interviewee**
Reduced AMU	“It's a bit difficult for us because we are not required to give antibiotics, if possible, these things should not happen.”	OTHER_007
	“I believe that right now, in my opinion, well in the beginning we used growth promoters in the past, antibiotic as growth promoters, but we are no longer using them.”	ASSOCIATION_02
Antibiotics still being used for growth promotion	“The government start to ban antibiotic in 2018. But I believe that now, when I visit the farms […] I always ask, “do you request the antibiotic”, “no, we still use”, especially in the starter phase.”	OTHER_09
	“To be honest, even we officially we banned the use of antibiotic in the feed since 2018, you know, some farmers still use it you know, and maybe secretly.”	OTHER_16
	“After all, the government has started to ban AGP [antibiotic growth promoters], but it's still being used by breeders. Because what, cheap. Replacing AGP is expensive.”	FARMER_09
Antibiotics not used for growth promotion even before ban	“Because by 2018 in Indonesia, the feed can't contain AGP, but in 2017 I was already non-AGP, I was already ahead of it.”	FARMER_07
	“I haven't used AGP for about 10 years.”	FARMER_09
Poorer AMU practices	“And the first time when the government make the policy, in 2018, for the antibiotic ban, the farmers buy antibiotics very easy and they put [administer antibiotics]. And they don’t know about the dosage, how much you add the antibiotic so they just give and give and give.”	OTHER_09
Reduced AMR	“Then what we feel the most is that by using a non-AGP, there is no resistance. If we use AGP, we have to do switching.”	FARMER_07
	“However, in the long term, this regulation might be better. Thus, the bacteria will be more sensitive to antibiotics. Also, many resistant bacteria are now due to the uncontrolled use of antibiotics. In the past, antibiotics could be used up to three kinds at a time. Now you can't have two categories. You can only have one type.”	OTHER_10

* AGP = antibiotic growth promoters, AMU = antimicrobial use, AMR = antimicrobial resistance.
