# Supplementary material for: “It’s a habit. They’ve been doing it for decades and they feel good and safe.”: A qualitative study of barriers and opportunities to changing antimicrobial use in the Indonesian poultry sector
Source: PLoS One. 2023 Sep 25;18(9):e0291556. doi: 10.1371/journal.pone.0291556 (PMC10519599; doi:10.1371/journal.pone.0291556)
Supplement: S3 Table — (PDF) [file pone.0291556.s005.pdf]

Supplementary Table S3: Summary of interviewees' opinions on the price and efficacy of feed additives as compared to antibiotic growth promoters*

Purpose of use	Opinion on price/efficacy	Excerpt	Interviewee
In response to drop in production	Not specified	“So when we see a decline in production then we will give them vitamins.”	FARMER_01
Improvement of feed quality<br>Routine use as replacement for antibiotics	Effective	“Yes, there are some manufacturers whose feed quality is really bad. But I handle that by giving the chickens prebiotics, enzymes and amino acid, sometimes antibiotics if I feel like there is bacteria in the chickens.”<br>“In my place, the use of antibiotics is minimal. Because for maintenance, I use enzymes, prebiotics, vitamins, amino acids, and others.”<br>“It [herbs] is effective. It can be consumed by the chicken, it can be sprayed into the litter.”	FARMER_02
When chickens are sick	Not specified	“Oh, we always provide vitamins. We mix vitamin C into the chicken feed.”	FARMER_04
Replacement for antibiotics	Not specified	“I have been using herbs for several years. Don't use antibiotics.”<br>“As long as I use the herbs, I think I almost don't use antibiotics at all.”	FARMER_05
Replacement for antibiotics	Not specified	“In principle for drugs, especially for antibiotics, we really try to minimize it. What we have on a regular basis are vitamins and additives such as amino acids and so on, that we use regularly. So we rarely prepare for antibiotics. We only use antibiotics if the chicken is really sick.”	FARMER_06
Replacement for antibiotics	Effective	“We use local herbal products, instead [of AGP]. We use curcuma and turmeric […] Yes, it is very effective and the ingredients are easy to access locally.”	FARMER_07
Improvement of feed quality	Not effective at preventing disease	“We switch from self-mixed feed to semi-finished feed. So we use concentrate. Of course, there are additional multivitamins and premixes which are added to increase the chicken's amino acid intake.”<br>“We have given drinking disinfectants, and the acidifier has also entered the chickens. But it can not help the chicken to survive against disease.”	FARMER_08
Replacement for antibiotics	More expensive than antibiotics<br>Similar efficacy	“Replacing AGP is expensive. There are organic acids, probiotics, it's so expensive.”<br>“Yes for the performance I think it’s [feed additives are] almost the same [as AGP]. But for healthy food I think we don't use AGP.”	FARMER_09
Drop in production<br>Replacement for antibiotics	Effective	“However, if suddenly it [the production] drops slightly, firstly I will give multivitamin, anti-stress or electrolytes then do the re-evaluation of what other actions is needed.”	FARMER_10

		"I think farmer can focus more on giving probiotic. For digestive system, probiotic is very good. Traditional medicine herbal is also good, but it is a long process."	
Supportive treatment (but not replacement of antibiotics)	Less expensive than antibiotics	"For example, the chicken has CRD [chronic respiratory disease], so we won't necessarily treat it immediately […] it turns out that the chicken will improve, then we at least can support with vitamins. But when the chicken is sick, we will still treat it with antibiotics."<br>"The cost [of antibiotics] is definitely higher, because usually [comparing] antibiotics with vitamins, antibiotics are more expensive."	MANAGER_04
Replacement for antibiotics	Cheaper than antibiotics	"We prefer to provide vitamins or amino acids that chickens need. While antibiotics are quite expensive and the price of chicken feed is also high, antibiotics will only add to the cost."	ASSOCIATION_04
Provided in feed to farmers upon request	More expensive than antibiotics<br>Less effective than antibiotics	"Because these alternatives first are much more expensive than antibiotics, secondly they are not as effective as antibiotics in terms of the growth of the chicken right, because they, actually before 2018 people don't use the antibiotics only to cure the disease, they use that to boost up the performance, right? And now it's, I think until now they still cannot solve the problem. I mean, there are some products that can be similar, the mechanism is similar but the result still cannot reach the level of antibiotics."	OTHER_04
Replacement for antibiotics	More expensive than antibiotics<br>Less effective than antibiotics	"As a comparison, actually before there was AGP, the production was indeed good, and the additional cost was small in economic calculation. Meanwhile, after the AGP is banned, the price of this AGP replacer can be up to 3 to 4 times the AGP, while the production is lacking."<br>"If they get the ingredients locally, [the cost of herbs is] not as high as AGP. But if the ingredients are imported, it's [the cost is] definitely above AGP."	OTHER_06
Replacement for antibiotics	More expensive than antibiotics	"For the essential oils, sometimes there are those who use them, and those who don't, because the cost is pretty expensive."	OTHER_07
N/A (not using self)	Less effective than antibiotics<br>More expensive than antibiotics	"If you want to change the antibiotic you have to add not only one, not only essential oil, or not only organic acid but also you have to combine […] When you use three kinds of the antibiotic replacer, the price is expensive."	OTHER_09
N/A (not using self)	More expensive than antibiotics<br>More effective than antibiotics	"The function of the herbs is actually to substitute the antibiotic growth promoters."<br>"But the price of the herbs, the extract is quite expensive if you compare to the price of the antibiotic growth promoters at this time."<br>"Even in some cases we found the production and the feed conversion efficiency is better when you use the herbs, especially we combine, if we combine some herbs, you know."	OTHER_16

* AGP = antibiotic growth promoters, AMU = antimicrobial use, AMR = antimicrobial resistance, CRD = chronic respiratory disease.
